# Advances in Biomechanical Parameters for Screening of Refractive Surgery Candidates: A Review of the Literature, Part III

**Published:** 2019

**Authors:** Majid Moshirfar, Mahsaw N. Motlagh, Michael S. Murri, Hamed Momeni-Moghaddam, Yasmyne C. Ronquillo, Phillip C. Hoopes

**Affiliations:** 1 Department of Ophthalmology and Visual Sciences, John A. Moran Eye Center, School of Medicine, University of Utah, Salt Lake City, UT, USA; 2 HDR Research Center, Hoopes Vision, Draper, UT, USA; 3 Department of Ophthalmology, College of Medicine, University of Arizona, Tucson, AZ, USA; 4 Department of Optometry, School of Paramedical Sciences, Mashhad University of Medical Sciences, Mashhad, Iran.

**Keywords:** Corneal Biomechanics, Ectasia, Refractive Surgery, Pentacam, Galilei, Corvis, Keratoconus

## Abstract

Corneal biomechanical properties have garnered significant interest in their relation to the development of ectatic corneal disease. Alongside the advent of corneal tomography and Scheimpflug imaging such as Pentacam and Galilei, there have been advances in assessing the cornea based on its biomechanical characteristics. Though the aforementioned imaging systems are highly capable of identifying morphologic abnormalities, they cannot assess mechanical stability of the cornea. This article, in contrast to Parts I and II of this article series, will focus on in vivo corneal biomechanical imaging systems. The two most readily available commercial systems include the Corvis ST and the Ocular Response Analyzer. Both of these systems aimed to characterize corneal biomechanics via distinct measurements. While in Parts I and II of this article series the authors focused on elevation, pachymetric, and keratometric data, the purpose of this article was to summarize biomechanical parameters and their clinical use in screening refractive surgery candidates. Moreover, this article explores biomechanical decompensation and its role in the development of corneal ectasia and keratoconus. There is a focus on the diagnostic accuracy of biomechanical indices in the identification of diseases such as keratoconus that may preclude a patient from undergoing refractive surgery.

## INTRODUCTION

**History and Background of Biomechanical Evaluation**

Parts I and II of this article series focused on single and dual Scheimpflug imaging. The advent of tomographic devices and the subsequent role three-dimensional corneal imaging in refractive screening have been well-documented in the literature. However, newer technologies that evaluate corneal biomechanics are newer and their role remains to be fully determined in corneal analysis. In the final part of our article series, we have elected to focus on biomechanical evaluation as it pertains to screening of the refractive surgical candidate.

Biomechanics is the study of mechanical laws as it pertains to the structural components of an organism or object. In the setting of biological tissues, the study of inherent material properties can help characterize function and facilitate understanding of factors that influence pathophysiology. The concept of corneal biomechanics has been a hot topic of research for several years. Since the 1960s, the viscoelastic structure of the cornea influences its mechanical properties [1, 2]. While ex vivo analysis of the corneal surface has been present for decades, it is only more recently that methods have been developed to study corneal biomechanics in vivo [3-7]. The inherent structural components of the cornea are altered in diseases such as ectasia and glaucoma. Moreover, changes in curvature, pachymetry, and elevation are all secondary signs of a biomechanically unstable cornea [8]. Thus, assessment of biomechanical properties can theoretically allow for better diagnosis and treatment of disease. 

**Figure 1:**
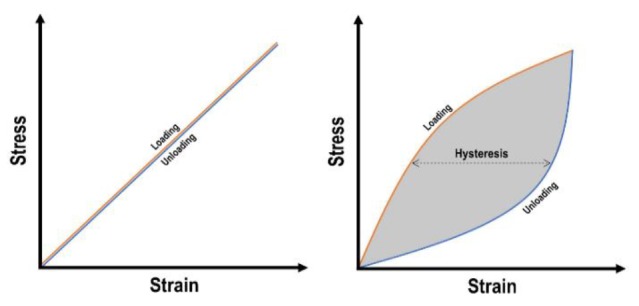
Stress-Strain Graph of a Linear Elastic Material (left) Compared to Viscoelastic Material (right). Hysteresis is Defined as the Shaded Area between the Loading and Unloading Curves

**Figure 2:**
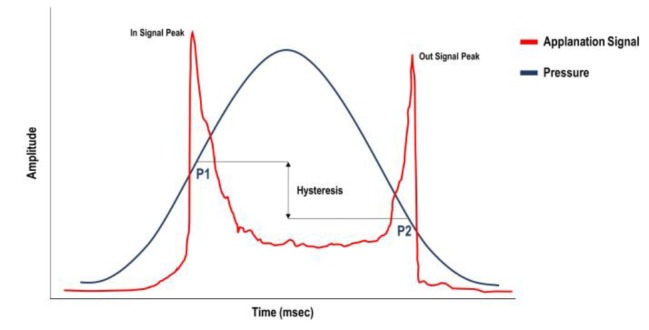
Signal Plot of Applanation Events on the Ocular Response Analyzer. Corneal Hysteresis is defined as the difference between Applanation Pressure at the first Event and Applanation Pressure at the Second Event. P1: Pressure at first Applanation; P2: Pressure at second Applanation

Biomechanics relies on principles of motion, momentum, and energy [3]. Important to our review is the general understanding that the cornea, like any other biological tissue, has a predictable non-linear, anisotropic, and inelastic behavior in response to stress and strain as shown in Fig. 1 [9]. Biomechanical analysis aims to evaluate corneal viscoelastic properties including hysteresis and stress relaxation. In addition, assessment of these properties allows for intraocular pressure (IOP) measurements to be less affected by corneal geometric characteristics and age, such that the IOP reading is as close as possible to the true IOP. There is significant interest in characterizing corneal biomechanical properties in the hopes of advancing screening methods for refractive surgery candidates. Currently, there are two commercially available devices that are capable of characterizing biomechanics in vivo: Corvis ST (CST: Oculus Optikgeraete GmbH; Wetzlar, Germany) and the Ocular Response Analyzer (ORA: Reichert, Buffalo, New York, USA). The ORA was first to reach the market in 2004 and provides data for hysteresis, resistance, pressure, and thickness, all with the intention of characterizing viscoelastic properties. The ORA consists of a rapid air impulse that applies force to the corneal surface and an advanced electro-optical system that monitors the corneal deformation response to the air impulse. It employs a noncontact tonometry (NCT) process, where a pulse of air lasting approximately 20 milliseconds is directed onto the corneal surface. This air pulse first flattens, or applanates, the corneal surface, measured at its first peak (P1) by the system’s collimation detector [10]. As the cornea relaxes to its natural convex shape following the symmetrical reduction of the air pulse there is a second applanation event, which again is measured at its peak (P2) [10]. The signal plot describing the applanation events is found in Fig. 2. The ORA utilizes the values of P1 and P2 to compute distinct corneal biomechanical parameters, found in Table 1. Recently the ORA software added the deformation signal waveform, which allows for a detailed morphologic description of corneal deformation [11-14]. The clinical application of these remains to be seen but has been the topic of several investigations [15-19]. The signal waveform is accompanied by several biomechanical parameters detailed in Table 2. 

**Figure 3:**
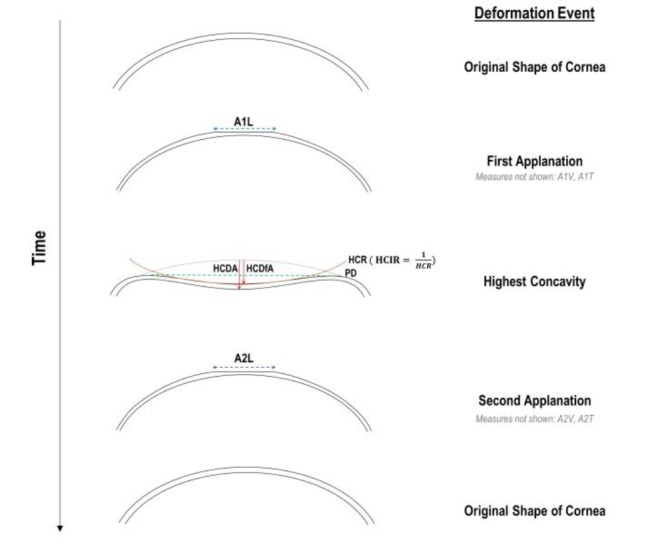
Corneal Deformation Events measured with the Corvis ST

Abbreviations: A1L: Length of Applanated Cornea at first Applanation; A1T: Time at first Applanation; A1V: Corneal Apex Velocity at first Applanation; A2L: Length of Applanated Cornea at second Applanation; A2T: time at second Applanation; A2V: Corneal Apex Velocity at second Applanation; HCDA: Deformation Amplitude at Highest Concavity; HCDfA: Deflection Amplitude at Highest Concavity; HCIR:Highest Concavity Inverse Radius of Curvature; HCR: Highest Concavity Radius of Curvature; PD: Peak Distance.

CST has currently been approved by the Food and Drug Administration (FDA) for tonometry and pachymetry. It also has the added benefit of a high-speed Scheimpflug camera that allows for in vivo characterization of the corneal biomechanical deformation response to an applied air pulse (constant metered collimated air pulse) [20]. The CST gathers over 4,000 frames per second within an 8 millimeter (mm) diameter along the horizontal corneal meridian. This device captures 140 images in 31 milliseconds after air pulse in the process of assessing the dynamic corneal response (DCR) parameters, IOP calculation, and corneal thickness measurements [21]. The CST reports a variety of biomechanical parameters visually correlated with the applanation events (Table 3 and Fig. 3). 

Both the ORA and CST are dynamic devices that allow for in vivo characterization of corneal biomechanics. Technologically, the key difference is that ORA adds a second P2 data point to become a bidirectional applanation device, while CST adds a Scheimpflug analyzer [22, 23]. In our review, we hope bring attention to and highlight the differences in terms of clinical application of these devices. Lastly, it is important to mention that the biomechanical evaluation of the cornea continues to evolve as the armamentarium of corneal analysis continues to grow. Methods still being tested include Brillouin optical microscopy, high-frequency ultrasound analysis, supersonic shear-wave technology, and swept-source optical coherence tomography (OCT) [3, 11, 24, 25]. While our review will primarily focus on the ORA and CST devices, it is important to consider these new technologies that will become integrated into future screening methods.

**Clinical Application in Corneal Analysis**

The structural integrity of the cornea is presumably disrupted in any underlying disease process. Abnormal biomechanical properties have been well documented in a variety of corneal diseases [8, 24]. Several diseases including floppy eyelid syndrome [26], pellucid marginal degeneration [27], glaucoma [28], diabetes mellitus [29, 30], and keratoconus [8] have been assessed with corneal biomechanics. Characterization of the cornea beyond the scope of pachymetry and topo/tomography can enhance the ability to identify disease [24]. Beyond the diagnostic vantage point, corneal biomechanics can provide a valuable quantitative assessment of the cornea that allows for risk-stratification of patients and predictive modeling of post-operative outcomes. Moreover, biomechanical analysis can help track treatment response and guide therapeutic management based on the level of disease severity.

Several studies have demonstrated the repeatability and precision of both the ORA and CST [21, 22, 31-40]. However, the majority of these studies have been conducted on normal eyes [22]. Further studies are required on pathologic eyes to validate the reproducibility and reliability of these devices for patients with suspected ectasia. 

**Table 1:** Description of Ocular Response Analyzer Output Parameters

Parameter	Description	Formula
CH	Assessment of the viscous-damping capacity of the cornea	P1 – P2
CRF	Assessment of overall corneal resistance	P1 – ĸP2
IOPcc	Ratio of P1 and P2 adjusted for biomechanical response of cornea	-
IOPg	Correlated with GAT, average of biphasic pressure measurements	(P1 + P2) / 2

Abbreviations: CH: Corneal Hysteresis; CRF: Corneal Resistance Factor; GAT: Goldmann Applanation tonometry; IOPcc: Corneal-compensated Intraocular Pressure; IOPg: Goldman-correlated Intraocular Pressure; P1: Pressure at First Applanation; P2: Pressure at Second Applanation; ĸ: Constant.

Nevertheless, biomechanical parameters are useful in diagnosis and management of many corneal diseases. For our review, we focus on keratoconus (KC) as the most common form of corneal ectasia, studied extensively in the literature [8, 18, 41-48]. Biomechanical decompensation seen in ectasia may be mainly the result of disruptions in the collagen matrix of the corneal stroma. In this article we present a comprehensive review of the literature for biomechanical evaluation of the cornea and its role in screening of the refractive surgery candidate. As with Part I and Part II of this article series, we use the collective term pre-keratoconus to replace the confusing and ambiguous terms of KC suspect, borderline KC, subclinical KC, form-fruste KC, and early KC. Through our analysis we aim to uncover the clinical application of biomechanics in distinguishing KC and pre-keratoconus and preventing the development of iatrogenic ectasia in patients undergoing refractive surgery.

**Table 2:** Description of Ocular Response Analyzer Corneal Deformation Waveform Parameters

Parameter	Description
aindex, bindex	Degree of non-uniformity and number of breaks in peak1 and peak2, respectively
p1area, p2area	Upper 75% area of applanation peak1 and peak2, respectively
aspect1, aspect2	Height/width aspect ratio for applanation peak1 and peak2, respectively
uslope1, uslope2	Rate of increase (slope) from 25% point of base to peak for peak1 and peak2, respectively
dslope1, dslope2	Rate of decrease (slope) from peak to 25% point of base for peak1 and peak2, respectively
w1, w2	Width of applanation peak at base for peak1 and peak2, respectively
h1, h2	Height of applanation peak from lowest to highest point of peak1 and peak2, respectively
dive1, dive2	Absolute value of length from peak until first break for peak1 and peak2, respectively
path1, path2	Absolute value of length around peak1 and peak2, respectively
mslew1, mslew2	Maximum increase in rise without a break for peak1 and peak2, respectively
slew1, slew2	Slope of dive1 and dive2, respectively
aplhf	High frequency noise between peak1 and peak2 normalized by average area
QI	Quality index for waveform selection for peak1 and peak2 (waveform score)
p1area1, p2area1	Upper 50% area of applanation peak1 and peak2, respectively
aspect1^1^, aspect2^1^	Height/width aspect ratio for 50% point of applanation peak1 and peak2, respectively
uslope1^1^, uslope2^1^	Rate of increase (slope) from 50% point of base to peak for peak1 and peak2, respectively
dslope1^1^, dslope2^1^	Rate of decrease (slope) from peak to 50% point of base for peak1 and peak2, respectively
w1^1^, w2^1^	Width of applanation peak at point of 50% of base for peak1 and peak2, respectively
h1^1^, h2^1^	Height of applanation peak from 50% point to highest point of peak1 and peak2, respectively
path1^1^, path2^1^	Absolute value of length around upper 50% of peak1 and peak2, respectively

**Table 3:** Corvis ST Biomechanical Parameter Descriptions

Deformation Phase	Parameter	Description
**Applanation 1**	A1DfA	Displacement of the corneal apex at first applanation in reference to initial state
	A1L	Length of flattened cornea at first applanation
	A1V	Maximum ingoing velocity at first applanation
	A1T	Time from initiation of air puff until first applanation
**Highest Concavity**	DA Ratio Max_1_	Ratio of deformation amplitude at corneal apex to deformation amplitude at points 1 mm peripheral to apex at highest concavity
	DA Ratio Max_2_	Ratio of deformation amplitude at corneal apex to deformation amplitude at points 2 mm peripheral to apex at highest concavity
	HCDA	Total corneal displacement at highest concavity
	HCDfA	Displacement of corneal apex at highest concavity in reference to initial state
	HCIR	Reciprocal value of radius of curvature at highest concavity
	HCR	Radius of curvature at highest concavity
	PD	Distance between the two peaks of the cornea at highest concavity
	T-HC	Time from initiation of air puff until highest concavity
**Applanation 2**	A2DfA	Displacement of the corneal apex at second applanation in reference to initial state
	A2L	Length of flattened cornea at second applanation
	A2V	Maximum outgoing velocity at second applanation
	A2T	Time from initiation of air puff until second applanation

Abbreviations: A1DfA: Deflection Amplitude at first Applanation; A2DfA: Deflection Amplitude at second Applanation; A1L: Length of Applanated Cornea at first Applanation; A2L= Length of Applanated Cornea at second Applanation; A1V: Corneal Apex Velocity at first Applanation; A2V: Corneal Apex Velocity at second Applanation; A1T: Time at first Applanation; A2T: Time at second Applanation; DA Ratio max1: Deformation Amplitude Ratio at 1 mm; DA ratio max2: Deformation Amplitude Ratio at 2 mm; HCDA: Deformation Amplitude at Highest Concavity; PD: Peak Distance; HCDfA: Deflection Amplitude at Highest Concavity; HCIR: Highest Concavity Inverse Radius of Curvature; HCR: Highest Concavity Radius of Curvature; T-HC: Time at Highest Concavity.

## METHODS

This literature review performed using various databases including PubMed, Mendeley, Ovid, Elsevier, and Science Direct. For the database search the primary search term included “corneal biomechanics”, connected to descriptors such as “Ocular Response Analyzer”, “Corvis ST”, “screening”, “keratoconus”, “subclinical keratoconus”, “mild keratoconus”, “form-fruste”, “biomechanical”, “waveform”, “metrics”, “index”, and various others. Peer-reviewed and scholarly resources including original scientific articles as well as review articles were included. Articles were screened for relevance and significance based on their abstracts. Those identified as appropriate for this review were included. Additional searches were made to find relevant literature through Mendeley, Ovid, Elsevier, and ScienceDirect. Publications between 1900 and 2019 were included. All articles deemed relevant to this topic were included. As with previous articles in this series, parameters with area under the curve (AUC) >0.900 were deemed suitable for screening of KC, while parameters with AUC>0.800 were selected for screening of pre-keratoconus. AUC was selected as the primary inclusion criteria as it inherently evaluates the diagnostic accuracy of a screening parameter. Indices that met these criteria in at least two studies were then averaged based on the cut-off value proposed by the individual study. The highlighted parameters in Tables 4 and 5 indicate the average selected cut-off values.

**Biomechanical Parameters for Refractive Screening**

Biomechanical failures are the primary abnormality in ectatic corneas [8]. The primary focus of this study is to define the specific biomechanical parameters studied in patients with KC. Based on the available literature, a side-by-side comparison of screening parameters with their respective sensitivity (SN), specificity (SP), and AUC for clinical KC and pre-keratoconus can be found in Tables 4 and 5. As we aim to identify how this data can enhance detection of corneal ectasia, we displayed the best screening indices along with our recommended cut-off values in Table 6. 

The following subsections detail the several indices available through ORA and CST available for screening. While these subsections aim to provide extensive detail with comparison of the literature regarding statistical power, we encourage the clinician to refer back to Table 4 and 5 for a visual summation of the data. The highlighted indices in these tables were selected for consideration of refractive screening. In comparison to Scheimpflug imaging, there are fewer investigations of corneal biomechanics. Nevertheless, based on our review, we recommend special attention to the indices of corneal hysteresis (CH), corneal resistance factor (CRF), Corvis biomechanical index (CBI), and tomographic and biomechanical index (TBI) as they are effective for the detection of pre-keratoconus.

**Table 4:** Summary of Biomechanical Parameters from Corvis ST and Ocular Response Analyzer in Detecting Frank Keratoconus

Study	Cut-off Value	Sensitivity	Specificity	AUC
ORA				
CH				
**Fontes et al [** 49 **]**	9.39	0.792	0.709	0.748
**Herber et al [** 50 **]**	9.40	0.800	0.800	0.868
**Mikielewicz et al [** 13 **]**	-	-	-	0.900
**Fontes et al [** 51 **]**	8.95	0.630	0.238	0.443
**Fontes et al [** 52 **]**	9.64	0.870	0.650	0.748
**Sedaghat et al [** 53 **]**	9.60	0.807	0.847	0.894
**Hallahan et al [** 54 **]**	8.50	0.520	0.954	0.748
**Touboul et al [** 42 **]**	9.60	0.660	0.670	0.680
**Hosseini et al [** 55 **]**	9.53	0.785	0.683	0.790
**Mohammadpour et al [** 56 **]**	8.75*	0.750	0.890	0.895
**Fontes et al [** 57 **]**	9.90	0.789	0.632	0.711
CRF				
**Fontes et al [** 49 **]**	8.68	0.779	0.756	0.767
**Herber et al [** 50 **]**	8.65	0.870	0.870	0.930
**Mikielewicz et al [** 13 **]**	-	-	-	0.968
**Fontes et al [** 51 **]**	7.40	0.283	0.405	0.341
**Fontes et al [** 52 **]**	9.60	0.905	0.660	0.770
**Sedaghat et al [** 53 **]**	8.90	0.855	0.891	0.946
**Hallahan et al [** 54 **]**	8.60	0.776	0.860	0.820
**Touboul et al [** 42 **]**	9.70	0.718	0.773	0.790
**Hosseini et al [** 55 **]**	8.75	0.803	0.764	0.820
**Mohammadpour et al [** 56 **]**	8.45*	0.900	0.930	0.966
**Fontes et al [** 57 **]**	8.90	0.684	0.789	0.737
DifCH				
**Galletti et al [** 47 **]**	-0.215	0.688	0.588	0.684
DifCRF				
**Galletti et al [** 47 **]**	-0.695	0.844	0.706	0.842
KMI				
**Herber et al [** 50 **]**	0.546	0.870	0.930	0.950
CST				
CBI				
**Herber et al [** 50 **]**	0.50	0.970	0.980	0.977
**Vinciguerra et al [** 58 **]**	0.50	0.941	1.000	0.982
**Vinciguerra et al [** 58 **]**	0.50	1.000	0.984	0.988
**Sedaghat et al[** 53 **]**	0.78*	0.966	0.993	0.998
**Ferreira-Mendes et al [** 45 **]**	0.085	0.783	0.933	0.893
**Ambrósio et al [** 59 **]**	0.49	0.946	0.975	0.977
**Steinberg et al [** 60 **]**	0.50	0.900	0.930	0.961
A1DfA				
**Chan et al [** 61 **]**	0.110	0.470	0.910	0.656
A2DfA				
**Chan et al [** 61 **]**	0.130	0.400	0.910	0.632
A1DfL				
**Chan et al [** 61 **]**	2.60	0.270	0.910	0.546
A2DfL				
**Chan et al [** 61 **]**	2.10	0.200	0.910	0.641
A1L				
**Chan et al [** 61 **]**	1.84	0.600	0.910	0.703
**Sedaghat et al [** 53 **]**	2.01*	0.759	0.818	0.837
**Elham et al [** 62 **]**	1.67	0.500	0.940	0.675
**Steinberg et al [** 63 **]**	1.771	0.340	0.340	-
A2L				
**Chan et al [** 61 **]**	1.34	0.530	0.910	0.573
**Sedaghat et al [** 53 **]**	0.97*	0.497	0.942	0.707
**Steinberg et al [** 63 **]**	1.824	0.320	0.320	-
A1V				
**Chan et al [** 61 **]**	0.19*	0.400	0.910	0.740
**Sedaghat et al [** 53 **]**	0.14	0.759	0.818	0.854
**Elham et al [** 62 **]**	0.12	0.688	0.640	0.692
A2V				
**Chan et al [** 61 **]**	-0.52*	0.670	0.910	0.787
**Sedaghat et al [** 53 **]**	-0.38	0.690	0.905	0.838
**Elham et al [** 62 **]**	-0.37	0.833	0.820	0.862
A1T				
**Chan et al [** 61 **]**	6.87	0.670	0.910	0.876
**Elham et al [** 62 **]**	7.03*	0.938	0.920	0.955
A2T				
**Chan et al [** 61 **]**	22.25	0.870	0.910	0.860
**Elham et al [** 62 **]**	21.4*	0.688	0.980	0.898
DA ratio max_1_				
**Herber et al [** 50 **]**	1.61*	0.880	0.880	0.951
**Ali et al [** 64 **]**	1.18	0.824	0.611	0.770
**Chan et al [** 61 **]**	1.64	0.870	0.950	0.937
**Tian et al [** 65 **]**	1.18	0.817	0.833	0.882
DA ratio max_2_				
**Herber et al [** 50 **]**	4.82*	0.880	0.980	0.958
**Chan et al [** 61 **]**	5.06	0.870	0.910	0.946
**Sedaghat et al [** 53 **]**	4.70	0.807	0.985	0.950
HCDA				
**Chan et al [** 61 **]**	1.19	0.730	0.910	0.863
**Sedaghat et al [** 53 **]**	1.10	0.676	0.781	0.784
**Elham et al [** 62 **]**	1.00*	0.729	0.940	0.893
HCDfA				
**Chan et al [** 61 **]**	0.96	0.800	0.910	0.829
HCDfL				
**Chan et al [** 61 **]**	5.73	0.130	0.910	0.521
**Steinberg et al [** 63 **]**	6.30	0.250	0.240	-
Integrated Radius				
**Herber et al [** 50 **]**	9.41*	0.900	0.930	0.974
**Sedaghat et al [** 53 **]**	8.70	0.876	0.934	0.961
HCIR				
**Herber et al [** 50 **]**	0.197*	0.920	0.930	0.962
**Chan et al [** 61 **]**	0.200	0.870	0.910	0.954
PD				
**Chan et al [** 61 **]**	5.25*	0.400	0.910	0.632
**Sedaghat et al [** 53 **]**	5.39	0.228	0.891	0.532
HCR				
**Chan et al [** 61 **]**	6.55	0.730	0.910	0.849
**Sedaghat et al [** 53 **]**	6.90*	0.897	0.861	0.939
**Elham et al [** 62 **]**	6.35	0.771	1.000	0.936
**Steinberg et al [** 63 **]**	6.899	0.260	0.250	-
SPA_1_				
**Herber et al [** 50 **]**	78.16	0.850	0.900	0.955
**Sedaghat et al [** 53 **]**	83.5*	0.862	0.949	0.965
T HC				
**Chan et al [** 61 **]**	17.40	0.270	0.910	0.576
TBI				
**Sedaghat et al [** 53 **]**	0.49*	1.000	1.000	1.000
**Ferreira-Mendes et al [** 45 **]**	0.385	0.971	0.981	0.998
**Ambrósio et al [** 59 **]**	0.79*	1.000	1.000	1.000

Abbreviations: A1DfA: Deflection Amplitude at first Applanation; A2DfA: Deflection Amplitude at second Applanation; A1DfL: Deflection Length at first Applanation; A2DfL: Deflection Length at second Applanation; A1L: Length of Applanated Cornea at first Applanation; A2L= Length of Applanated Cornea at second Applanation; A1V: Corneal Apex Velocity at first Applanation; A2V: Corneal Apex Velocity at second Applanation; A1T: Time at first Applanation; A2T: Time at second Applanation; AUC: Area under the Curve; CBI: Corvis Biomechanical Index; CH: Corneal Hysteresis; CRF: Corneal Resistance Factor; CST: Corvis ST; DA Ratio Max1: Deformation Amplitude Ratio at 1 mm; DA Ratio Max2: Deformation Amplitude Ratio at 2 mm; DifCH: Corneal Thickness-Corrected Corneal Hysteresis; DifCRF: Corneal Thickness-Corrected Corneal Resistance Factor; HCDA: Deformation Amplitude at Highest Concavity; HCDfA: Deflection Amplitude at Highest Concavity; HCDfL: Deflection Length at Highest Concavity; HCIR: Highest Concavity Inverse Radius of Curvature; HCR: Highest Concavity Radius of Curvature; KMI: Keratoconus Match Index; ORA: Ocular Response Analyzer; PD: Peak Distance; SPA1: Stiffness Parameter at first Applanation; TBI: Tomographic and Biomechanical Index; T HC: Time at Highest Concavity.*denotes cut-off point with best area under the curve if more than one study evaluated index accuracy. Highlighted indices represent the parameters included in the final evaluation of proposed thresholds.

**Table 5:** Summary of Biomechanical Parameters from Corvis ST and Ocular Response Analyzer in Detecting Pre-Keratoconus

Study	Cut-off Value	Sensitivity	Specificity	AUC
ORA				
Aspect1				
**Luz et al [** 66 **]**	15.69	0.571	0.605	0.614
Aspect1^1^				
**Luz et al [** 66 **]**	23.78	0.619	0.461	0.540
Aspect2				
**Luz et al [** 66 **]**	13.423	0.571	0.671	0.630
Aspect2^1^				
**Luz et al [** 66 **]**	16.56	0.524	0.724	0.617
CH				
**Johnson et al [** 67 **]**	9.10	0.690	0.913	0.854
**Labiris et al [** 68 **]**	9.80*	0.885	0.880	0.904
**Luz et al [** 66 **]**	9.90	0.571	0.513	0.607
**Galletti et al [** 69 **]**	9.19	0.726	0.655	0.710
**Kirgiz et al [** 70 **]**	9.45	0.760	0.760	0.850
CRF				
**Hashemi et al [** 71 **]**	8.60	0.870	0.853	0.920
**Johnson et al [** 67 **]**	9.10	0.714	0.896	0.847
**Labiris et al [** 68 **]**	8.90*	0.890	0.932	0.931
**Luz et al [** 66 **]**	8.90	0.619	0.684	0.622
**Galletti et al [** 69 **]**	8.46	0.808	0.724	0.840
**Kirgiz et al [** 70 **]**	9.25	0.880	0.880	0.900
DifCH				
**Ruiseñor Vázquez et al [** 72 **]**	-0.215	0.781	0.552	0.699
**Galletti et al [** 69 **]**	-	-	-	0.700
DifCRF				
**Ruiseñor Vázquez et al [** 72 **]**	-0.695	0.863	0.713	0.848
**Galletti et al [** 69 **]**	-0.77*	0.849	0.736	0.850
Dive1				
**Luz et al [** 73 **]**	279	0.619	0.628	0.647
**Luz et al [** 66 **]**	279*	0.619	0.631	0.649
Dive2				
**Galletti et al [** 69 **]**	205.8*	0.699	0.678	0.740
**Luz et al [** 66 **]**	230.75	0.571	0.461	0.554
Dslope1				
**Luz et al [** 66 **]**	26.39	0.667	0.487	0.599
Dslope1^1^				
**Luz et al [** 66 **]**	35.21	0.476	0.447	0.501
Dslope2				
**Luz et al [** 66 **]**	16.73	0.524	0.697	0.622
Dslope2^1^				
**Luz et al [** 66 **]**	30.65	0.619	0.526	0.604
H1				
**Luz et al [** 73 **]**	319.68*	0.619	0.692	0.667
**Luz et al [** 66 **]**	319.69	0.619	0.684	0.663
H1^1^				
**Luz et al [** 73 **]**	213.12*	0.619	0.692	0.667
**Luz et al [** 66 **]**	213.13	0.619	0.684	0.663
H2				
**Galletti et al [** 69 **]**	277.8*	0.740	0.621	0.740
**Luz et al [** 66 **]**	262.69	0.524	0.671	0.629
H2^1^				
**Hashemi et al [** 71 **]**	190*	0.870	0.918	0.940
**Galletti et al [** 69 **]**	185	0.740	0.621	0.740
**Luz et al [** 66 **]**	175.13	0.524	0.671	0.629
KMI				
**Labiris et al [** 68 **]**	0.721	0.857	0.875	0.940
Mslew1				
**Luz et al [** 66 **]**	89.0	0.571	0.645	0.622
Mslew2				
**Galletti et al [** 69 **]**	111.5*	0.575	0.770	0.700
**Luz et al [** 73 **]**	95.5	0.524	0.705	0.643
**Luz et al [** 66 **]**	20.73	0.619	0.526	0.622
Path2				
**Luz et al [** 66 **]**	25.23	0.429	0.461	0.505
Path2^1^				
**Luz et al [** 66 **]**	35.03	0.619	0.474	0.564
P1-area				
**Luz et al [** 73 **]**	2968.5*	0.667	0.603	0.714
**Luz et al [** 66 **]**	2885.19	0.667	0.658	0.707
P1-area1				
**Luz et al [** 73 **]**	1301.5*	0.762	0.539	0.721
**Luz et al [** 66 **]**	1237.5	0.714	0.632	0.717
P2-area				
**Galletti et al[** 69 **]**	1968.0*	0.658	0.667	0.700
**Luz et al [** 66 **]**	20.13.0	0.571	0.605	0.597
P2-area1				
**Galletti et al [** 69 **]**	817.6*	0.644	0.701	0.710
**Luz et al [** 66 **]**	884.5	0.571	0.553	0.566
Slew1				
**Luz et al [** 66 **]**	56.36	0.619	0.540	0.558
Slew2				
**Luz et al [** 66 **]**	274.13	0.619	0.632	0.629
Uslope1				
**Luz et al [** 66 **]**	54.714	0.619	0.540	0.627
Uslope1^1^				
**Luz et al [** 66 **]**	57.0	0.524	0.461	0.557
Uslope2				
**Luz et al [** 73 **]**	65.5	0.571	0.692	0.641
**Luz et al [** 66 **]**	65.5*	0.571	0.697	0.642
Uslope2^1^				
**Luz et al [** 66 **]**	46.58	0.524	0.750	0.622
CST				
A1T				
**Peña-García et al [** 74 **]**	7.46	0.500	0.799	0.736
A1L				
**Steinberg et al [** 63 **]**	1.775	0.380	0.380	-
AL2				
**Catalán-López et al[** 75 **] **	1.48	0.610	0.220	0.690
**Steinberg et al [** 63 **]**	1.832	0.330	0.330	-
CBI				
**Kataria et al [** 76 **]**	0.01	0.680	0.770	0.725
**Ferreira-Mendes et al [** 45 **]**	0.005	0.772	0.679	0.775
**Ambrósio et al [** 59 **]**	0.07*	0.681	0.823	0.822
DA Ratio Max_1_				
**Peña-García et al [** 74 **]**	1.09	0.536	0.793	0.775
PD				
**Catalán-López et al [** 75 **]**	4.93	0.750	0.510	0.670
HCR				
**Catalán-López et al [** 75 **]**	7.52	0.750	0.500	0.680
**Steinberg et al [** 63 **]**	7.231	0.400	0.400	-
SPA_1_				
**Kataria et al [** 76 **]**	93.74	0.660	0.830	0.745
TBI				
**Kataria et al [** 76 **]**	0.16	0.840	0.860	0.850
**Ambrósio et al [** 77 **]**	-	0.933	0.924	0.932
**Ambrósio et al [** 77 **]**	-	1.000	0.992	0.999
**Ferreira-Mendes et al [** 45 **]**	0.295	0.895	0.910	0.960
**Ambrósio et al [** 59 **]**	0.29*	0.904	0.960	0.985
**Koc et el [** 78 **]**	0.29	0.670	0.860	0.790
**Chan et al [** 79 **]**	0.16	0.844	0.824	0.925

**Abbreviations: A1L: Length of Applanated Cornea at first Applanation; A2L= Length of Applanated Cornea at second Applanation; A1T: Time at first Applanation; AUC: Area under the Curve; CBI: Corvis Biomechanical Index; CH: Corneal Hysteresis; CRF: Corneal Resistance Factor; CST: Corvis ST; DA ratio max**_1:_ **Deformation Amplitude Ratio at 1 mm; DifCH: Corneal Thickness-Corrected Corneal Hysteresis; DifCRF: Corneal Thickness-corrected Corneal Resistance Factor; HCR: Highest Concavity Radius of Curvature; KMI: Keratoconus Match Index; ORA: Ocular Response Analyzer; PD: Peak Distance; SPA**_1_ : Stiffness Parameter at first Applanation; TBI: Tomographic and Biomechanical Index;*denotes cut-off point with best area under the curve if more than one study evaluated index accuracy. Highlighted indices represent the parameters included in the final evaluation of proposed thresholds **. Please refer to Table 2** **for defination of other included terms in this table.**

**Table 6:** The Biomechanical Parameter Clinical “Cheat Sheet”: Suggested Cut-off Values for Keratoconus Indices in Screening Clinical Keratoconus and Pre-Keratoconus

Parameter	Clinical Keratoconus	Pre-Keratoconus
	**Cut-Off Value**	**Cut-Off Value**
ORA		
CH	-	9.45
CRF	8.67	8.86
DifCRF	-	-0.733
CST		
CBI	0.55	-
DA Ratio Max_1_	1.63	-
DA Ratio Max_2_	4.86	-
HCR	6.90	-
HCIR	0.199	-
Integrated Radius	9.06	-
SPA_1_	80.8	-
TBI	0.56	0.23

Abbreviations: CBI: Corvis Biomechanical Index; CH: Corneal Hysteresis; CRF: Corneal Resistance Factor; CST: Corvis ST; DA ratio max1: Deformation Amplitude Ratio at 1 mm; DA ratio max2: Deformation Amplitude Ratio at 2 mm; DifCRF: Corneal Thickness-corrected Corneal Resistance Factor; HCIR: Highest Concavity Inverse Radius of Curvature; HCR: Highest Concavity Radius of Curvature; ORA: Ocular Response Analyzer; SPA1: Stiffness Parameter at first Applanation; TBI: Tomographic and Biomechanical Index.

**Ocular Response Analyzer**

***Corneal Hysteresis***

Hysteresis refers to the energy dissipation that occurs during a stress-strain cycle, demonstrated in Fig. 1 [36, 80]. The cornea exhibits hysteresis as a result of its component materials, namely collagen that allows for a loss or dampening of energy when stress is applied. Biomechanical systems measure CH as the energy absorbed during the applanation process [81]. For the ORA system, CH is measured as the difference between the two applanation events, which is equivalent to P1 minus P2 in millimetre of mercury (mmHg) (Fig. 2) [22]. While CST also measures biomechanical properties, its approach to deformation analysis involves different parameters described in subsequent sections.

Energy absorption during corneal deformation results in different speeds during the inward and outward applanation peaks. Thus, CH aims to quantify the viscoelastic mechanical damping effect of the cornea as measured by the difference between these applanation pressures [82]. In recent validation studies, the mean normal values of CH have been reported between 10.0-11.0 mmHg [46, 82, 83]. However, this range is plagued with significant variability well-documented in the literature [22].

Nevertheless, CH values are significantly lower in ectatic corneas compared to normal, healthy eyes [77, 84, 85]. Shah and colleagues were the first groups to quantitatively compare CH in healthy and keratoconic eyes. While their study demonstrates significant differences between these two populations, they also reported significant overlap in CH ranges between healthy and ectatic corneas that makes it an unreliable parameter in diagnosis of KC [46, 86]. As seen in Tables 4-6, a multitude of subsequent studies has confirmed the conclusion that CH as a standalone parameter is not capable of clearly distinguishing frank KC or pre-keratoconus [42, 43, 50, 51, 53-57, 66, 67, 73, 87]. There are significant differences in CH measurements between ectatic and healthy corneas; however, this parameter does not have adequate diagnostic accuracy [47].

There are a few studies [13, 88] that reported diagnostic credibility for CH, which we define as AUC >0.900. However, these conclusions have been brought into question regarding confounding parameters that may influence diagnostic accuracy of CH. This is supported by a growing body of evidence that documents CH to be heavily influenced by baseline factors such as degree of myopia, central corneal thickness (CCT), age, IOP, corneal curvature, corneal temperature, corneal hydration, the location and area of the applied force, and also the speed and pressure of the air pulse during the loading and unloading phase [89], which hinders its validity and diagnostic accuracy [22, 90-92]. Thus, taking this into consideration, the studies controlled for these intrinsic factors were more likely to demonstrate poor diagnostic accuracy for CH [57]. As CCT increases, diagnostic accuracy of CH decreases; in fact, for CCT ≥ 520 µm it was noted particularly poor predictive value [57].

Despite its limitation as an individual refractive screening parameter, CH is a useful index to assess biomechanical function and baseline characteristics. CH was identified as a helpful parameter in differentiating astigmatic corneas from those that may have pre-KC [70]. While CH alone did not have sufficient diagnostic accuracy, its AUC of 0.850 lends reassurance to its value as an adjunct parameter in identifying patients at-risk of iatrogenic ectasia. CH also has a role in monitoring treatment outcomes. that CH values are significantly altered following surgical procedures such as surface ablation or laser-assisted in-situ keratomileusis (LASIK) [41, 93, 94], which may assist in identifying therapeutic response to treatment.

A recent study proposed correction factors using regression analysis for CH to improve its diagnostic value [69]. While this has enhanced its performance as a refractive screening parameter, it requires further external validation. Based on our review we do not recommend the use of CH as an individual parameter for screening of the surgical candidate. However, as demonstrated in Tables 4-6, there is diagnostic value and importance of CH that has yet to be fully realized. We recommend its clinical use in a multivariate index or as an adjunct parameter that complements clinical evaluation.

***Corneal Resistance Factor***

CRF is an ORA parameter that aims to quantify the overall viscoelastic resistance of the cornea with an emphasis on the elastic properties of the cornea [82, 95]. Specifically, CRF is derived from the formula (P1 – ĸP2), where ĸ is a constant determined from analysis of P1, P2, and CCT [1, 3]. Similar to CH, the mean normal values of CRF have been reported 10.0-11.0 mmHg [46, 82, 83], with significant differences documented between healthy and diseased corneas[96]. Further, CRF is similar to CH in regards to its vulnerability to baseline factors that influence its diagnostic accuracy [22, 90-92]. However, given its strong correlation to CCT [10], CRF is a more robust predictive index and this is supported by many studies demonstrated its excellent predictive accuracy in discriminating frank KC [50, 53, 71] (Table 5). It is also important to disclose there are several studies demonstrated CRF as a poor diagnostic parameter [42, 43, 51, 55-57, 87]. The inconsistencies in the literature are likely influenced by selection criteria, population demographics, and the aforementioned baseline confounding factors. Based on our review, CRF is better suited for discrimination of frank KC than CH but its use alone for diagnosis is not recommended.

Studies evaluating the diagnostic value of CRF in pre-keratoconus are limited. While some studies demonstrate excellent predictive accuracy [13, 70, 71], others do not corroborate these findings and recommend against using CRF for diagnosis of pre-keratoconus [66, 67, 69, 73, 86]. The lack of uniform conclusion limits the use of CRF alone, but also promotes its value as a helpful adjunct parameter for screening.

Recently, a corneal thickness-corrected CRF (DifCRF) reported that maintained a moderate diagnostic accuracy (AUC=0.848) for detection of pre-keratoconus [72]. That findings were similar to the study [47] that also employed DifCRF. Through logistical regressions, the diagnostic accuracy modestly improved (AUC = 0.878), but still failed to demonstrate excellent predictive accuracy as seen in Table 6 [72]. Interestingly, CRF with a waveform parameter was combined and reported a 100% SP [71]. This reiterates the value of multivariate indices and the additive predictive value when combining screening parameters.

***Intraocular Pressure***

For the purposes of our review, we have provided a section on IOP as there is a growing body of evidence that suggests IOP can influence other biomechanical parameters and conversely, biomechanical properties can also affect IOP measurements [97, 98]. While the gold-standard of ophthalmic instruments for IOP assessment is the Goldmann applanation tonometer (GAT), several studies have evaluated the ability of the ORA to accurately measure IOP. The ORA calculates Goldmann-correlated (IOPg) and corneal-compensated (IOPcc) estimates of IOP (Table 1). IOPg is calibrated to match the measurements made through Goldmann tonometry [10, 95]. IOPcc incorporates a specific ratio of the P1 and P2 pressures adjusted for the biomechanical response of the cornea [10].

The overwhelming majority of studies compared ORA estimates to the reference GAT calculations have found that the ORA slightly overestimates IOP [82, 94, 99, 100]. However, there are also a handful of studies that find no significant difference between the IOP measurements and instead report excellent reproducibility of IOP measurements [40, 101, 102]. This discrepancy indicates that further comparative studies are required to reach a definitive conclusion regarding IOP precision with ORA. Moreover, the studies investigated IOP as a screening parameter have indicated that IOPg and IOPcc are not strong parameters in distinguishing KC or pre-keratoconus [44, 77]. Instead, both parameters may serve as complementary measurements for detection of disease [86]. However, identify IOPg as a parameter capable of discerning KC [53, 70]. These preliminary findings warrant further investigation with external validation. At this point, however, we do not recommend the use of IOP for screening of refractive surgery candidates. Nevertheless, it is important to mention for the purpose of completeness and understanding of the ORA system.

***Keratoconus Match Index and Keratoconus Score***

The Keratoconus Match Index (KMI), which is also known as the Keratoconus Score (KS), is a parameter provided by the ORA that represents the probability of existing KC based on a normative database[50]. KMI evaluates seven waveform scores, described below, through an applied neural network [103]. The studies evaluated KMI, while limited, demonstrate it may have clinical use in discriminating KC [50, 68, 103]. It may also have a future role in staging KC based on ORA analysis as demonstrated by the recent study [104]. However, similar to IOP, we have mentioned this parameter for completeness and do not recommend its routine use for diagnosis of KC or pre-KC.

***Waveform Analysis***

Waveform-derived parameters were recently introduced in the ORA software. These variables are related to characteristics of the applanation signal such as width, peak, and height and are summarized in Table 2 [77]. There are identifiable differences in waveform morphology between diseased and healthy corneas. Multiple studies report that applanation in signal curves in KC are more likely to contain oscillations, lower amplitudes, and more variability [19, 87, 95, 105].

Beyond the morphological differences, waveform parameters have been studied in discriminating KC. However, there are different conclusions regarding which waveform parameter is best-suited for screening. For example, several studies have corroborated the importance of p2area, a parameter that represents the area of the second peak in the waveform plot [13, 66]. More recently, dive, which quantifies the backside downslope of each peak, was identified as the best waveform parameter for discriminating KC [18]. In another recent study, waveform parameter was concluded H2^1^ as a highly predictive index, particularly in cases of pre-KC [71]. While other parameters did not share the predictive accuracy H2^1^, the majority of waveform indices were significantly different between eyes with pre-KC and healthy controls. Further studies are required to extrapolate the diagnostic value of these indices and whether a combination index would prove valuable in screening surgical candidates.

Some studies have also developed novel indices based on the existing waveform parameters. High sensitivity and specificity were reported with a novel index based on existing topographic gold standards and waveform analysis [106]. Similarly, higher predictive accuracy in a novel index reported that indirectly measures maximum deformation amplitude via a minimum infrared signal [54, 107].

Regardless of the selected parameter, there is no dispute that ORA waveform signals provide additional information that supplements screening for both KC and pre-KC [84]. In fact, waveform analysis may be superior to measurements of CH and CRF for screening of ectasia [66, 73]. This is supported by a recent case, that reported a case of unilateral ectasia in which CH and CRF were nearly equal in both eyes, but waveform morphology was significantly different [19].

A newer feature on the ORA is the Waveform Score (WS), which is a quantitative analysis of the ORA measurement signal based on seven individual waveform parameters. The proprietary algorithm presents WS as a value from 0 to 10. The higher the score, the more reliable are the ORA metrics. However, waveform signal, and thus WS, is a direct function of the individual cornea being analyzed and may thus not be suitable for reliable screening of KC. Nonetheless, it is an important measurement in its identification of the most reliable waveform signal, which in effect can vastly influence the assessment of other waveform parameters.

The applanation signal curve and waveform parameters are highly valuable in clinical assessment of the cornea. Similar to other ORA indices, it is difficult to recommend a particular index for screening purposes as there are inconsistencies in the literature regarding its predictive value. Nevertheless, there is agreement that waveform-derived parameters are beneficial to pre-operative screening and should be analyzed in each patient undergoing biomechanical analysis with ORA.

**Corvis ST**

***Dynamic Corneal Response Parameters***

As shown in Table 3, the CST provides several dynamic corneal response parameters (DCR) in response to the various phases of corneal deformation. Briefly, we will define and review these parameters as it pertains to the events of that occur during the deformation process and a graphical representation is available in Fig. 3. At the time point of the first applanation, the applanation length (A1L), defined as the length of the applanated segment, is measured. In addition to the applanation length, the corneal apex velocity is measured at both the first and second applanation events (A1V and A2V, respectively) as well as the time at first applanation (A1T).

At the time of maximum concavity, several parameters are measured: deflection amplitude (HCDfA), highest concavity deflection length (HCDfL), deformation amplitude (HCDA), highest concavity radius (HCR), maximum inverse radius (HCIR), and peak distance (PD). HCDfA refers to the displacement of the corneal apex in reference to the cornea in its initial state. This should not be confused with HCDA, measure as the sum of corneal deflection amplitude plus whole eye movement. In simpler terms, HCDA is the total translational movement of the cornea in the anterior-posterior direction [95]. HCR is more straightforward and is defined as the radius of the cornea at the maximum concavity state based on a parabolic best-fit curve [108]. PD refers to the distance between the two peaks of the cornea in the maximum concavity state [108].

The reciprocal value of HCR defines the maximum inversive radius (HCIR) [109]. Related to HCIR is a newer parameter called the integrated radius [110], calculated as the integral of the AUC of the inverse concave radius.

At the time of second applanation, many parameters are measured that largely echo those analyzed during the first applanation event. The primary indices are applanation length (A2L), time at second applanation (A2T), and the corneal apex velocity toward second applanation (A2V).

**Figure 4:**
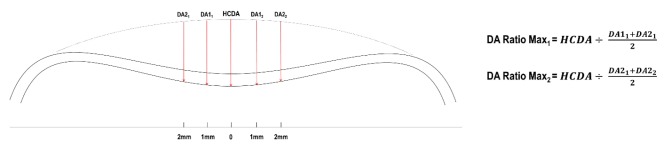
Calculation of Deformation Amplitude Ratio at 1mm and 2 mm

Abbreviations: DA11: Deformation Amplitude at 1 mm to the Left of the Central Apex, DA12: Deformation Amplitude at 1 mm to the Right of the Central Apex, DA21: Deformation Amplitude at 2 mm to the Left of the Central Apex, DA22: Deformation Amplitude at 2 mm to the Right of the Corneal Apex, DA ratio max1: Deformation Amplitude Ratio at 1 mm, DA ratio max2: Deformation Amplitude Ratio at 2 mm, HCDA: Highest Concavity Deformation Amplitude; mm= Millimeter.

Two relatively new parameters include the deformation amplitude ratio (DA ratio) and the deflection amplitude ratio (DfA ratio). The DA ratio is calculated as the deformed amplitude of the central apex divided by the average deformation of two points located 1mm (DA ratio max_1_) or 2 mm (DA ratio max_2_) on either side of the apex, (Fig. 4). Similarly, the DfA ratio is calculated as the ratio between the deflection amplitude divided by the amplitude of two points located 1 mm or 2 mm peripherally from the corneal apex. The lower the ratio, the more resistant the cornea is to deformation/deflection. Conversely, in ectatic corneas that are not as stiff, the ratios are expectedly higher. The studies evaluated DCRs for frank KC and pre-KC corneal apex as shown in Table 4 and 5.

Similar to the biomechanical parameters of the ORA, DCRs are vulnerable to confounding effects of IOP, pachymetry, and age [108, 111, 112]. Maximum keratometry (K) also frequently affects DCR measurements [50]. Some studies have investigated this relationship, like Vinciguerra and colleagues who reported that the parameters most immune to the influence of these confounders were HCR, HCIR, DA ratio, and DfA ratio [108]. Furthermore, normative values were defined based on subgroups.

As demonstrated in Tables 4 and 5, the DCRs have a valuable clinical role in screening patients for KC. However, there is no consensus regarding which DCR is superior. While some studies have identified A1V as the best parameter in differentiating KC [113, 114], others have identified integrated radius or DA ratio [53, 61, 79]. Moreover, the recent study demonstrated that all DCR indices are capable of distinguishing KC [50].

Some studies have taken the approach of controlling for CCT [62], which we consider the best approach for study design when evaluating DCRs. Other studies like have shown that DCRs only marginally improve KC diagnosis and cannot currently be considered standalone parameters for screening purposes [63]. For pre-KC, there is no clear conclusion based on the literature. The overall trend indicates that no single parameter provides sufficient discriminatory power to distinguish pre-KC [75].

Ultimately, we recommend CST parameters deserve clinical attention and play a valuable role in biomechanical assessment of KC, but further studies are required to determine which indices are most appropriate for refractive screening. Beyond refractive screening, DCRs can also be useful in monitoring biomechanical changes following photorefractive keratectomy (PRK) and LASIK [115, 116]. However, the lack of consensus points to limitations in existing studies. For patients with pre-keratoconus we continue to recommend a multi-faceted approach that evaluates DCRs but does not rely on them alone for screening.

**Corvis Biomechanical Index**

First described, the CBI is based on a linear regression analysis of dynamic corneal response parameters measured by the CST in combination with the corneal horizontal thickness profile [58]. CBI is calculated using a logistic regression analysis with DA ratio at 1 and 2 mm, first applanation velocity, the standard deviation of deformation amplitude at highest concavity, the Ambrosio relational thickness to the horizontal profile (ARTh), and a novel stiffness parameter [116]. In this seminal study, near perfect discriminatory power was demonstrated with an AUC of 0.988 for diagnosis of KC [58]. Subsequent validations have confirmed the excellent predictive power of CBI in identifying frank KC [53], demonstrated in Table 4. However, external validation studies for identification of pre-keratoconus remains limited [45, 110, 117]. Despite promising initial results shown in Table 5, there is a need for further research to validate its diagnostic credibility in these patients.

More recently, the adjusted CBI (aCBI) was proposed, a modified parameter that eliminates corneal thickness data from the linear regression analysis [118]. Interestingly, aCBI showed higher diagnostic accuracy compared to the original CBI [60]. The aCBI was studied on relatively small sample size (n=29) and lacked an external validation dataset [118]. Nevertheless, corneal biomechanics alone may be able to discern KC. However, at this time we recommend the use only of CBI for screening of patients prior to refractive surgery. While CBI is capable of discerning frank KC with ease, we recommend using it with caution and in combination with other clinical data for identification of pre-KC.

**Intraocular Pressure**

As described in the section on ORA parameters, it is important to discuss IOP as it pertains to biomechanical parameters especially as it can influence assessment and measurements [97, 98]. CST provides two IOP measurements: noncorrected IOP (IOPnct) and a recently introduced biomechanically corrected IOP (bIOP) [119]. bIOP is a unique measurement that estimates IOP based on an algorithm that reduces the confounding effects of age and stiffness parameters [108, 119].

CST has demonstrated highly reproducible and precise measurements of IOP [21, 32, 64]. Moreover, the CST measured values for IOP have no statistical difference when compared to gold-standard measurements of GAT [21]. There is evidence to suggest CST may also underestimate IOP, which may delay measurements in diseases such as glaucoma that rely on pressure measurements for clinical monitoring [21]. Unlike the ORA system, there are minimal studies in the literature evaluating CST IOP measurements for discrimination of ectasia. Despite its limited role in refractive screening, we have included this section for completeness.

**Stiffness Parameters**

Recently novel stiffness parameters were introduced, defined as the resultant pressure, or loading force, at the first inward applanation divided by corneal displacement [109]. In their seminal paper they introduced stiffness parameter at applanation 1 (SPA1) and stiffness parameter at highest concavity (SPHC), which aim to quantify corneal resistance to deformation. While the resultant pressure used in each of these parameters is equal, the displacement value differs. SPA1 is calculated using the displacement between the apex of the undeformed cornea and the deflection at A1 [109]. On the other hand, SPHC uses the displacement between the corneal position at A1 and maximum deflection at highest concavity [109]. The stiffness parameters are inherently a function of IOP because resultant pressure is calculated as the air pressure from applanation minus the IOP. 

These novel parameters were the first quantifiable indices that allowed for interpretation of DCRs in relation to corneal deformation and stiffness. However, only SPA1 has been validated in subsequent studies, and even these investigations are limited [50, 53, 76]. We have included these studies in Tables 5 and 6 to raise awareness for the clinicians of the available parameters. Due to the lack of validation studies, we do not recommend its use in screening but recognize the stiffness parameters are valuable adjunct parameters for understanding of corneal stiffness and intrinsic biomechanics.

**Tomographic and Biomechanical Index**

The TBI is a recently introduced parameter based on a robust combination of biomechanical and Scheimpflug-based tomographic data from the CST and Pentacam HR (Oculus Optikgerate GmbH, Wetzlar, Germany) [107]. TBI is highly accurate in detecting frank KC as demonstrated in Table 4 [53]. Perhaps more importantly, TBI combines both tomographic and biomechanical data along with artificial intelligence to optimize detection of subtle changes of pre-KC, demonstrated in several studies [45, 59].

TBI demonstrated superior diagnostic accuracy when compared to CBI and Belin/Ambrósio enhanced ectasia total deviation value (BAD_D) [76]. This is similar to results found in the initial study that introduced TBI [59]. These impressive results have also been replicated in studies [45, 79]. However, a recent study did not confirm this diagnostic credibility in patients with pre-keratoconus [78]. While TBI had the highest AUC among tested parameters, it was still short of diagnostic value with an AUC of 0.790. Admittedly, however, this was a small population (n = 21) which may have skewed the results based on intrinsic baseline factors, selection criteria, or population demographics.

While TBI is capable of discerning pre-KC, it should be used with caution to ensure appropriate selection of refractive surgery candidates. It is more accurate than previously analyzed indices in the detection of pre-KC and provides the unique advantage of sensitively identifying patients with normal topography who may otherwise be missed when screening for surgical eligibility.

## DISCUSSION

**Application and Interpretation of Biomechanical Indices**

Early detection of KC remains a clinical challenge. Currently, most diagnostic and classification criteria for KC and pre-KC is based on anterior curvature data derived from Scheimpflug-based or slit-scanning systems [120-123]. Given the growing consensus that KC may begin as focal thinning as a result of biomechanical instability [8, 124, 125], it is warranted to consider in vivo biomechanical assessment as an appropriate approach for screening of corneal ectasia. Based on our current understanding of pathophysiology, changes in biomechanical properties may occur before disease becomes apparent via tomography or topography. Furthermore, we recommend the use of CH and CRF through ORA along with CBI and TBI through CST when screening for pre-KC. This recommendation is based on a comprehensive review of the available literature but should not be considered a diagnostic guideline in evaluation of the surgical candidate. Rather, we encourage the clinicians to use the cut-off values in Table 6 as supportive evidence when there is already a high index of suspicion for pre-KC.

Moreover, based on our review there are several clinical applications of biomechanical parameters. It can not only assist in screening eligible surgical candidates but can also track post-operative changes with the hope of preventing iatrogenic ectasia [126]. Our hope is that this review can serve as a quick reference guide alongside the clinical decision-making process of the individual surgeon. Moreover, we encourage the use of biomechanical assessments in the context of the larger clinical picture. The biomechanical data should be considered with patient history, physical exam, and anterior curvature data amongst others. Through a combined approach we can best care for the patient and offer appropriate therapies and treatment.

**Limitations**

As in vivo biomechanical assessment is relatively new approach there are several limitations to discuss. First, since CST and ORA are two independent instruments with different output parameters, we cannot investigate their agreeability. To this end, while CST employs a fixed pressure air pulse, the ORA uses a variable pressure dependent on the value of P1. Thus, each system has a different loading force applied to the surface that can impact the biomechanical assessment of the cornea. In addition, we do not have a current understanding of a biomechanical model that describes the “normal” cornea. Without a baseline understanding of normal physiological assessment, it is difficult to truly evaluate the biomechanical instability that contributes to pathogenesis of ectasia.

Another important limitation to consider is the confounding effect of age, CCT, IOP, and K reading amongst others. These baseline intrinsic factors influence biomechanical assessment [50, 74, 108, 127]. It is important to control for these factors, yet many of the current studies available in the literature do not address this important point in study design. We recommend eliminating confounding effects through stratification and linear transformation.

Other extrinsic factors may also influence biomechanical assessment, such as ocular hydration which varies throughout the time of day. In fact, there was a significant reduction in highest concavity time in dry eyes compared to normal eyes [128]. Beyond dry eye, there are studies documented alterations in biomechanics in the setting of diabetes mellitus, hypertension, contact lens wear, ethnicity, and degree of myopia [29, 129-134]. Understanding the relationship between these factors and biomechanical instability will be important for optimization of screening protocols and methods.

In general, one of the limitations to consider with both ORA and CST is that the surface they applanate is too large to identify subtle changes in biomechanical properties [135]. As a result, it is difficult to identify the exact changes in biomechanical parameters or in the corneal stiffness after treatments like corneal cross-linking (CXL) using the instruments available in the clinic. This limits the quantitative assessment of treatment response when considering an expected increase in biomechanical rigidity after long-term treatment with CXL [135]. The only available method of reliably documenting these biomechanical changes is through confocal microscopy [136-138]. Nevertheless, experts in biomechanical analysis like Vinciguerra have shown that new DCRs may be capable of identifying biomechanical changes following CXL [110, 139]. Among the new CST parameters, the integrated inverse concave radius was the only parameter that showed a significant decrease in the four years follow-up after CXL, which was consistent with stiffening [138]. Given the inconsistencies in the available literature, future studies should incorporate large sample sizes to overcome this limitation and determine the utility of ORA and CST in monitoring therapeutic success of treatment.

**Looking Ahead**

The future of refractive screening relies on a combined approach with a multivariate index. By incorporating tomographic and biomechanical variables, we can enhance the ability to distinguish early forms of disease from normal, healthy eyes [140]. A more comprehensive screening to differentiate between normal and suspicious corneas can be performed using different indices such as BAD_D index and the newly developed CBI and TBI indices [141]; screening corneal objective risk of ectasia (SCORE) analyzer (Bausch & Lomb, Rochester, NY, USA) which incorporated several parameters in the calculation of the SCORE including inferior-superior (I-S) asymmetry, corneal irregularity at 3 mm zone, thinnest corneal pachymetry, the difference between central and thinnest pachymetry (CP - TP), decentration of the thinnest point along the vertical meridian, maximum posterior elevation, anterior elevation of the thinnest point and the pachymetric thinning rate [142]; percentage similarity of the examined cornea with abnormal corneas using the Zeiss Atlas 9000 PathFinder II Corneal Analysis Software (Humphrey Atlas, Carl Zeiss Meditec AG, Jena, Germany) which combines the quantitative parameters including the corneal irregularity measurement (CIM), the mean toric keratometry (MTK) and shape factor (SF) [143]; and assessment of the epithelial, and stromal thickness map patterns with OCT [144].

Combination indices can improve screening of both frank KC and pre-KC [66, 76, 145]. Studies that incorporate biomechanical data have superior diagnostic accuracy and the additional data from Scheimpflug-based tomography plays an essential role in screening surgical candidates. Recently, the Brazilian Artificial Intelligence on Corneal Tomography and Biomechanics (BrAIn) proposed a combination index that successfully discriminated pre-KC with high sensitivity (AUC=0.945) [146]. By integrating tomography data with biomechanical parameters, their study shows enhanced screening methods. Combining discriminant functions aids in the biomechanical detection of pre-KC, but the study showed an optimal AUC of 0.893, which is just shy of diagnostic standards [74]. Interestingly, this study controlled for IOP and CCT while the BrAIn study did not, which may explain the discordant findings. Regardless, these studies are an indication of the future direction of refractive screening. It is also the reason we recommend using available combination indices such as CBI and TBI when the information is available.

Future studies should consider the value of epithelial thickness profiles and incorporate Fourier domain optical coherence tomography (OCT) to assess the early changes of pre-KC. Several studies have already demonstrated that tomographically normal eyes can still manifest significant differences in epithelial thickness [144, 147-153]. With better understanding of the various modalities, we can enhance the discriminatory power of refractive screening even further. The use of OCT, Brillouin microscopy, and epithelial thickness mapping may also improve diagnostic parameters for tracking progression of KC [125, 147, 154-156]. As such, we encourage future studies to consider the important complementary tests available beyond biomechanical assessment.

## CONCLUSION

Biomechanical evaluation of the cornea is undoubtedly helpful in both understanding the pathophysiology of corneal disease and in evaluating refractive surgery candidates. The advent of in vivo characterization by Corvis ST and Ocular Response Analyzer allows for direct analysis of biomechanics with a particular purpose of screening for keratoconus and pre-KC. However, the role of biomechanical evaluation in the clinical setting remains to be fully defined. Moreover, the lack of conclusive evidence regarding pre-KC diagnosis makes it a clinical challenge based on biomechanical parameters alone. This holds true for other approaches of corneal analysis including Scheimpflug imaging, tomography, and OCT amongst others. Standalone parameters have not been validated and require further investigation. Fortunately, different approaches for interpretation are rapidly developed and may result in concrete screening methods in the future. For the time being, this review reiterates the importance of considering combined refractive indices in differentiating healthy versus diseased eyes.

## DISCLOSURE

Ethical issues have been completely observed by the authors. All named authors meet the International Committee of Medical Journal Editors (ICMJE) criteria for authorship of this manuscript, take responsibility for the integrity of the work as a whole, and have given final approval for the version to be published. No conflict of interest has been presented.

## Funding/Support:

None
